# Intrinsic Functional Brain Architecture Derived from Graph Theoretical Analysis in the Human Fetus

**DOI:** 10.1371/journal.pone.0094423

**Published:** 2014-05-01

**Authors:** Moriah E. Thomason, Jesse A. Brown, Maya T. Dassanayake, Rupal Shastri, Hilary A. Marusak, Edgar Hernandez-Andrade, Lami Yeo, Swati Mody, Susan Berman, Sonia S. Hassan, Roberto Romero

**Affiliations:** 1 Merrill Palmer Skillman Institute for Child and Family Development, Wayne State University, Detroit, Michigan, United States of America; 2 Department of Pediatrics, Wayne State University School of Medicine, Detroit, Michigan, United States of America; 3 Perinatology Research Branch, NICHD/NIH/DHHS, Bethesda, Maryland and Detroit, Michigan, United States of America; 4 Department of Neurology, University of California at San Francisco School of Medicine, San Francisco, California, United States of America; 5 Basic Medical Sciences Program, Wayne State University School of Medicine, Detroit, Michigan, United States of America; 6 Department of Psychiatry and Behavioral Neurosciences, Wayne State University, Detroit, Michigan, United States of America; 7 Department of Obstetrics and Gynecology, Wayne State University School of Medicine, Detroit, Michigan, United States of America; 8 Department of Radiology, Wayne State University School of Medicine, Detroit, Michigan, United States of America; Max Planck Institute for Human Cognitive and Brain Sciences, Germany

## Abstract

The human brain undergoes dramatic maturational changes during late stages of fetal and early postnatal life. The importance of this period to the establishment of healthy neural connectivity is apparent in the high incidence of neural injury in preterm infants, in whom untimely exposure to ex-uterine factors interrupts neural connectivity. Though the relevance of this period to human neuroscience is apparent, little is known about functional neural networks in human fetal life. Here, we apply graph theoretical analysis to examine human fetal brain connectivity. Utilizing resting state functional magnetic resonance imaging (fMRI) data from 33 healthy human fetuses, 19 to 39 weeks gestational age (GA), our analyses reveal that the human fetal brain has modular organization and modules overlap functional systems observed postnatally. Age-related differences between younger (GA <31 weeks) and older (GA≥31 weeks) fetuses demonstrate that brain modularity decreases, and connectivity of the posterior cingulate to other brain networks becomes more negative, with advancing GA. By mimicking functional principles observed postnatally, these results support early emerging capacity for information processing in the human fetal brain. Current technical limitations, as well as the potential for fetal fMRI to one day produce major discoveries about fetal origins or antecedents of neural injury or disease are discussed.

## Introduction

A major objective for neuroscience is to build a complete diagram of brain connections at the beginning of human life. Functional MRI has recently proven capable of measuring neural connectivity in the human fetal brain [1], [2]. By leveraging correlations of low-frequency (<∼0.1 Hz) intrinsic fluctuations in the blood oxygen level dependent (BOLD) signal, functional connectivity MRI (fcMRI) provides information about macroscale brain organization. While this method is based on functional signals, intrinsic brain fluctuations have been shown to reflect underlying anatomic pathways [3], [4], making this a useful technique for exploring emergent neural circuits in the human fetus.

Graph theory is a method used in mathematics to extract global organizational principles for physical, biological, or social systems by modeling interrelations between units or members of the system. For example, airline flight routes, migration patterns, social networks, and Twitter feeds all may be studied using graph theory. This technique conveys information about overall network infrastructure as well as specific features, such as which ‘nodes’ (locations/individuals) within a system are central ‘hubs’ of connectivity, linking numerous other units to one another. In the past several years, graph theory-based approaches have proven highly effective for defining organizational structure of human brain networks (reviewed by [5]). For example, from graph analysis of fMRI datasets we have learned that the human brain is organized with small world topology [6] and that the posterior cingulate and insular cortices are connectivity hubs [7]–[9].

Graph analysis of fMRI data involves first dividing the brain into a set of distinct predefined regions from which BOLD timeseries are extracted, then correlating timeseries with one another in a pairwise fashion, yielding an *n*×*n* correlation matrix. Here, each *a priori* selected brain region, or *n*, is a ‘node’, while each matrix value is an ‘edge’ denoting the relationship for a given pair. Edges are reported either as weighted with positive/negative connection strength above a threshold, or as binary (present, absent). Diverse metrics exist for gleaning topological network organization from brain connectivity matrices (see [5]), but it has been suggested that when numerous edges within the graph are negative, modular assessment is a preferred approach [10].

Modularity measures the degree to which a network can be partitioned into non-overlapping subsets of regions (“modules”) that are internally interactive, while sparsely interactive with outside areas. Within a modular network, critical nodes (brain regions) serve specific roles integrating local connections (intramodular hubs) or branching between modules (intermodular connectors [11]). First applied to adults [12]–[14], and later to children [15], [16] and infants [17], [18], graph-based fMRI analyses show that the human brain has modular architecture.

Prior work has highlighted age-related differences in module size and composition [e.g., 19, 20] demonstrating the utility of tracing the profile of modular architecture across development. As children mature, brain network configuration shifts from a local to a distributed organization. That is, characteristic functional brain subnetworks or “modules” emerge wherein spatially distant brain regions such as frontal and parietal regions, comprising the executive control network, and between medial prefrontal and parietal regions, comprising the default mode network, become more strongly connected [21].

Graph-based MRI analyses are relevant to cognition [22], and can differentiate unique patterns of atypical connectivity in neurodevelopmental disorders such as attention deficit-hyperactivity disorder (ADHD [23]), autism [24], [25], and Tourette’s syndrome [26]. Overall, graph analysis has proven valuable in understanding human brain organizational structure, developmental processes, and disease sequelae. However, it is unknown whether recognizable principles of human functional brain organization (i.e. modular organization) are evident before birth. The present study utilizes a graph theoretical analysis to elucidate the functional architecture of the human fetal brain.

## Materials and Methods

### Enrollment

Participants were recruited while receiving prenatal care at clinics located in Hutzel Women’s Hospital. Physicians provided initial orientation to the study. Interested and eligible women were introduced to members of the research team who provided a study overview. This included introduction to MRI technology, a study timeline, and a review of the consent form. Exclusion criteria included history of claustrophobia or contraindications for MRI. In addition, the topic of safety and fetal MRI was discussed prior to enrollment. For a review of safety and fetal fMRI, please refer to prior works that have addressed this topic in more detail [27], [28]. Women were informed that their participation in this research was voluntary and not related to their clinical care. They were assured that they may discontinue involvement at any time and that personal information would remain confidential.

### Participants

Thirty-eight pregnant women (singleton, uncomplicated pregnancies, median maternal age = 24 years, range = 18 to 34 years) underwent MRI examination within the 19^th^ to 39^th^ week of pregnancy. The median gestational age (GA) of their fetuses was 31 weeks (range = 19+6 to 38+5, weeks+days) at the time of MRI. Five participants were excluded prior to group level analyses due to fewer than 90 fMRI volumes retained after removing high movement frames, leaving a total of 33 participants. fMRI data from 19 of these participants has been reported previously [2].

### Ethics Statement

Written informed consent to undergo MRI was obtained from all participants. The Human Investigation Committee of Wayne State University approved the study protocol.

### Image Acquisition

Participants underwent MRI examination using a Siemens Verio 70-cm open-bore 3-T MR system using a 550 g abdominal 4-Channel Siemens Flex Coil. Resting-state fMRI data were collected with the following echo planar imaging (EPI) BOLD parameters: repetition time/echo time (TR/TE), 2000/30 ms; 4 mm slice thickness, no skip; 25 axial slices; 80° flip angle. Real-time adjustments were made to imaging protocols during scan acquisition; in cases where data were cut off, image artifacts were present, or movement levels were appraised as high, additional BOLD fMRI data were collected. As a result, the total number of fMRI time fames obtained varied from 180 to 463, 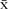 = 343, across participants. The total imaging protocol was restricted to a maximum of 45 minutes duration.

### Functional Data Preprocessing

Fetal data were preprocessed using methods adapted from our prior work [2]. In brief, preprocessing included manual segmentation of the fetal brain from surrounding tissue, manual reorientation, volume realignment, normalization to a 32-week fetal template [29] retaining native resolution (3.44×3.44×4 mm), segment realignment, and smoothing.

### Movement

Multiple steps were performed to address image motion-related artifacts. First, only low movement time frames were included in analyses. This strategy draws on the fact that although motion in the fetus is substantial, there are periods of relative stability (e.g., <1.5 mm) for which conventional volume motion correction would be expected to suffice. Participants retaining fewer than 90 volumes after frame exclusion were excluded from analyses. Next, volume realignment was performed using SPM8. Following this, mean framewise displacement across the scan and root mean square (RMS) were calculated and averaged for translational (*T_x_, T_y_, T_z_*) and rotational (*R_pitch_, R_roll_, R_yaw_*) movement. Lastly, translational and rotational movement parameters (with another six parameters representing their first order temporal derivatives) were removed with covariate regression analysis before computing ROI pairwise correlations. Two-sample t-tests were used to compare translational and rotational movement parameters between younger and older fetal groups (younger, GA <31 weeks, n = 17; older GA≥31 weeks; n = 16). Any movement parameter that was found to be significantly different between age groups was included as covariate in partial correlations assessing correspondence between age and graph theoretical measures. Movement data were processed with SPSS version 21, using a p≤0.05 significance level.

### Spectral Clustering for Region of Interest (ROI) Identification

A spatially constrained group level clustering approach [30] was used to generate spatially contiguous ROIs that are evenly sized and distributed across the cortex, including the cerebellum, and excluding cerebral spinal fluid (CSF). Briefly, this method produces functionally homogenous clusters by assessing voxel timeseries similarity using Pearson correlations, then iteratively merges voxels whose within-cluster similarity is maximal and between-cluster similarity is minimal. Next, it identifies the most representative clusters of voxels using a normalized cut algorithm [6] and performs group level clustering. This method produces ROIs that are optimally functionally homogenous and consistent across individuals. Fetal fMRI data were gray matter masked and analyzed to generate 150 ROIs. These were then classified by hemisphere, by lobe, and by coordinates corresponding to center of mass. One of the 150 ROIs was anomalous (spatially discontiguous) and was removed from analysis. Resultant fetal ROI files are available at www.brainnexus.com for download.

### Participant Connectivity Matrices

**Figure 1:**
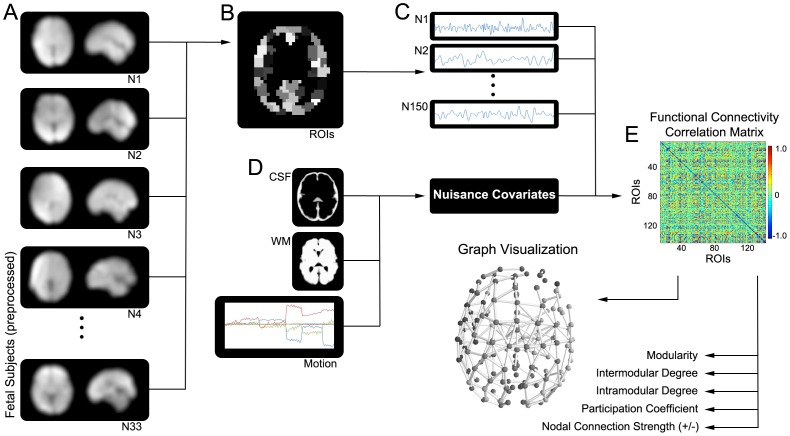
Fetal fMRI graph processing pipeline. 33 preprocessed fetal fMRI datasets (**A**) were divided into 149 distinct regions of interest (**B**), from which regional blood oxygen level dependent (BOLD) timeseries was extracted (**C**). After removing principal components of signals from cerebrospinal fluid (CSF), gray matter (GM), and white matter (WM), as well as the translational and rotational movement parameters (**D**), timeseries were correlated with one another in pairwise fashion to derive a 149×149 correlation matrix (**E**), which was used to assess graph metrics (e.g., modularity) and to derive graph visualizations of human fetal brain networks.

The CONN-fMRI Toolbox ver.12.p [31] was used to measure correlations between ROIs. Rather than removing the global signal to reduce spurious noise effects, we used the anatomical component correction (aCompCor) method of estimating and removing noise [32], [33]. Principal components of signals from white matter and cerebral spinal fluid, as well as translational and rotational movement parameters (with another six parameters representing their first order temporal derivatives), were removed with covariate regression analysis (see **Figure 1**). Pearson’s correlation coefficients were then estimated from time series data for each pair of regions. Fisher’s transformation was used to convert coefficients to z-scores to produce FC correlation matrices for each participant. Correlation matrices were entered into network analyses using methods described in the following section.

### Graph Processing

The Brain Connectivity Toolbox (https://sites.google.com/site/bctnet/) was used to compute fundamental graph theory metrics. Graphs were examined in their unthresholded, weighted state with negative weights included using the Rubinov-Sporns algorithm to determine the optimal modular partitioning of each participant’s network [10]. We investigated both global and regional topological properties of brain graphs. At the global level, we assessed modularity (*Q*), which measures the degree to which each network can be clearly separated into minimally overlapping modules by comparing the ratio of within-module edges (connections) and between-module edges in the observed graph versus the ratio expected by chance. A highly modular network is one in which subsets of nodes have many connections between them and few connections to the remaining nodes in the network. Because the modularity algorithm is heuristic and produces minimally varied partitions from run to run, 1000 iterations were run in order to identify the partition that was most consistently identified across runs. The selected partition was then subjected to a fine-tuning algorithm to further optimize the modularity [10]. Pearson’s correlations were used to test for associations between GA and modularity (*Q*) and intermodular mean correlation strength.

We assessed two basic nodal properties: positive connection strength and negative connection strength. We then assessed six nodal properties based on each node’s pattern of connections within its own module and to the remaining modules. These were: (1) positive participation (correlation) coefficient, (2) negative participation coefficient, (3) within module positive strength Z score, (4) within module negative strength Z score, (5) between module positive strength Z score, and (6) between module negative degree Z score. Participation coefficients reflect the fraction of a node’s edges that connect to other nodes within the same module. *Within* module strength z-score is calculated as the summed weight of positive edges connecting a node to other nodes within the same module. This value, the within-module strength, is then scaled by the mean within-module strength for all other nodes in the same module to obtain the corresponding z-score. *Between* module strength z-scores are calculated in the exact same fashion, except only counting edges between a node and all other nodes belonging to other modules. Robust regression tested the relationship of age and each nodal measure. Fetuses were divided at median age 31 weeks to facilitate comparison of modularity structure in older versus younger fetal groups. P values were corrected for multiple comparisons using false discovery rate correction with p≤0.05 (as described by [34]).

Next, we evaluated the relation between length and strength of connections across ROI pairs. For each participant, correlation strengths obtained for every pairwise comparison (N = ∼11,000) were organized into 20 bins of size.1, covering the full range from −1 to 1. Euclidean distance between each ROI pair was computed. Average connection length in each correlation strength bin was determined for each participant. Two sample t-tests were used to compare the strength of the 5% longest connections and 5% shortest connections between older and younger fetuses using an alpha value of p = 0.025.

## Results

Birth outcomes for newborns that were scanned *in utero* as participants in this study are provided in **Table 1**.

**Table 1:** Summary of Participant and Data Characteristics.

	Younger Fetuses (n = 17)		Older Fetuses (n = 16)		
	Mean	SD	Mean	SD	p-value
GA at MRI (weeks)	27.6	2.88	34.4	2.31	***<0.001*** *
GA at birth (weeks)	39.0	1.14	39.0	1.16	0.93
Birth weight (g)	3195.6	325.05	3423.0	578.11	0.18
Neonatal length (cm)	49.5	1.98	50.6	3.33	0.26
Head circumference (cm)	33.6	1.67	34.7	2.14	0.12
Mean translational movement (mm)	0.38	0.13	0.48	0.17	0.08
Mean rotational movement (radians)	0.77	0.49	1.16	0.62	***0.05*** *
Translational RMS movement (mm)	0.28	0.07	0.29	0.07	0.77
Rotational RMS movement (radians)	0.01	0.00	0.01	0.00	0.34
	**Male**	**Female**	**Male**	**Female**	**p-value**
Sex	9	7	11	6	0.62
	**Median**	**IQR**	**Median**	**IQR**	**p-value**
Apgar at 1 minute	9	1	9	1	0.55
Apgar at 5 minutes	9	0	9	0	0.37

Younger fetuses are defined as GA <31 weeks, older fetuses are defined as GA≥31 weeks.

*denotes significant p-values. Abbreviations: GA, gestational age; MRI, magnetic resonance imaging; M, male; F, female; SD, standard deviation; IQR, interquartile range.

### Data Summary

On average 343 frames, or 11.6 minutes of fMRI data, were collected for the 33 participants included in our analysis. After removing high movement frames, we retained 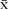 = 208 frames for each participant, or 60.6% of the total data collected. A larger number of fMRI frames were removed in younger compared to older fetuses (younger 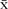 = 168, SD ±58; older 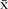 = 104, SD ±50; p = 0.001), and older fetuses retained a larger number of fMRI frames for analyses (younger 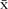 = 187, SD ±60; older 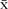 = 228, SD ±45; p = 0.03). The average duration that fetal data were collected continuously without interruption by movement was 33 consecutive frames, or ∼1 minute. The total number of interruptions introduced into consecutive data acquisition by excluding high movement segments was 5.9 (S.D. = 2.1), including breaks between scans. Specific absorption rate (SAR) is a measure of the heating caused by radio-frequency energy deposition in the body. Here, average SAR across fMRI volumes was 0.24 W/kg (S.D. = 0.08), well within FDA safety exposure limits (3 W/kg/10-minute exposure).

### Movement

Translational and rotational movement for younger and older fetal groups are summarized in **Table 1**. Movement values were on average <1 millimeter/radian, and thus within accepted standards (cf. [23]). Younger and older fetal groups did not differ in translational or rotational framewise displacement, or RMS, nor did they differ in mean translational movement. However, fetal age groups showed a significant difference in mean rotational movement (p = 0.053), and as a result, mean rotational movement was included as a covariate in all subsequent age-related global analyses.

### Modularity

Intermodular mean connection strength was positively correlated with GA, r = 0.4 p = 0.02. This result indicates that with development, functional modules become more tightly integrated into a whole brain system. There was a related negative relationship between modularity (*Q*; mean = 0.35, standard deviation = 0.1, range: 0.14–0.6, normally distributed) and GA, r = −0.38 p = 0.03. This suggests that after correction for movement variation, modularity decreases with increasing GA. Higher modularity, as observed in younger fetuses, is indicative of more segregated functional subnetworks.

**Figure 2:**
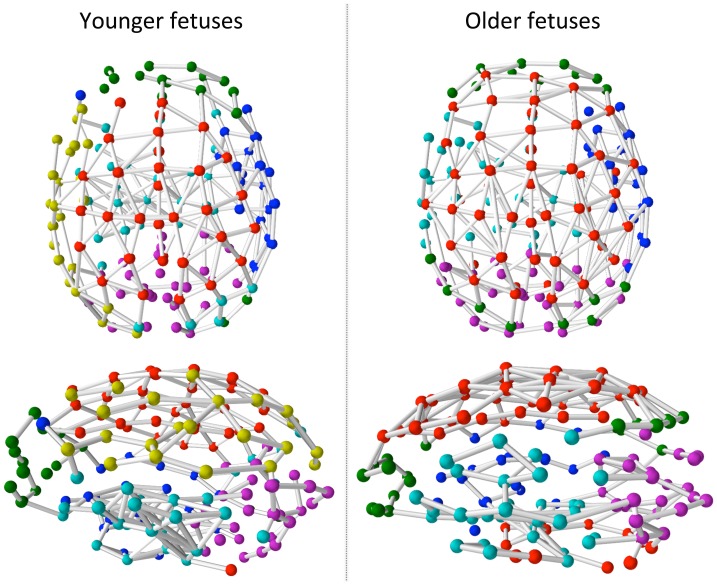
Macro-scale functional brain network modules for younger ( = 27.6 weeks; n = 17) and older ( = 34.4 weeks; n = 16) fetuses. Nodes are represented as colored spheres and edges are represented as the connections between nodes. Common colors are assigned to maximally similar modules in the two groups, but color assignments are arbitrary.

**Figure 3:**
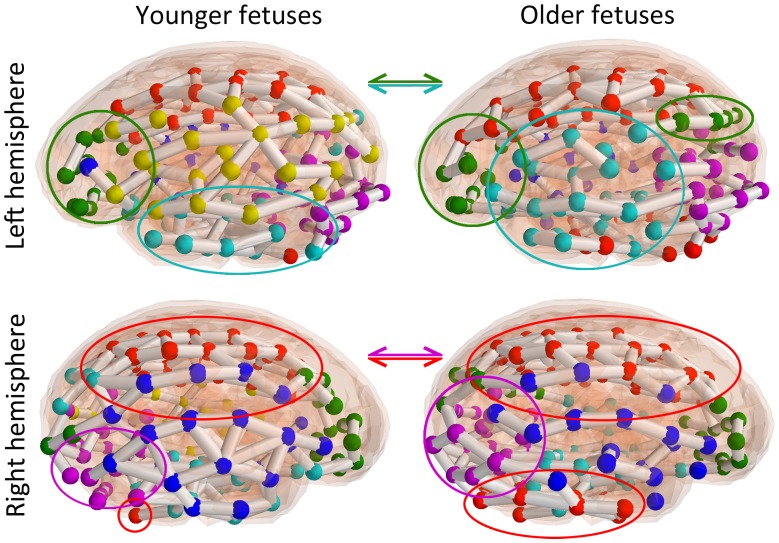
Specific modular changes with development in younger versus older fetuses. Graph visualizations provided in Figure 2 are depicted for the right and left lateral brain perspective and overlaid on a semi-transparent anatomical reference scan. Modules that show significant reconfiguration in older fetuses are highlighted. In older fetuses, sensorimotor cortices (red nodes, red circles) were more functionally connected to regions of the cerebellum and inferior temporal cortices. Prefrontal areas (green nodes, green circles) were more functionally connected to regions of the posterior parietal cortex in older fetuses. A ventral frontal temporal cortex module (teal nodes, teal circles) was present in both groups, but this module became more left lateralized and functionally integrated with inferior frontal and parietal regions in older fetuses. The occipital module (magenta nodes, magenta circles) expanded superiorly to encompass primary and secondary visual cortices in older fetuses.

While modules derived for younger and older fetuses showed similarities in their topological organization (e.g., the right hemisphere temporal insular cortex module was similarly distributed in both groups; **Figure 2**, blue), differences were noted for several modules. For example, sensorimotor cortices (**Figure 3**, red nodes, red circles) appear more functionally connected to regions of the cerebellum and inferior temporal cortices in older fetuses. Additionally, prefrontal areas (**Figure 3**, green nodes, green circles) appear more functionally connected to regions of the posterior parietal cortex in older fetuses. Furthermore, a ventral frontal temporal cortex module (**Figure 3**, teal nodes, teal circles) was present in both groups, but this module appeared to became more left lateralized and functionally integrated with frontal and inferior parietal regions in older fetuses. Only in older fetuses did this module encompass areas that will develop into Broca’s and Wernicke’s Areas, critical for speech comprehension and production. Finally, the occipital module (**Figure 3**, magenta nodes, magenta circles) appeared to better encompass the boundaries of primary and secondary visual cortex in older compared to younger fetuses. We also discovered a left, lateral parietal and premotor cortical module (**Figure 3**, yellow nodes) that was more prominent only in younger fetuses.

### Strength and Length of Functional Brain Connections

**Figure 4:**
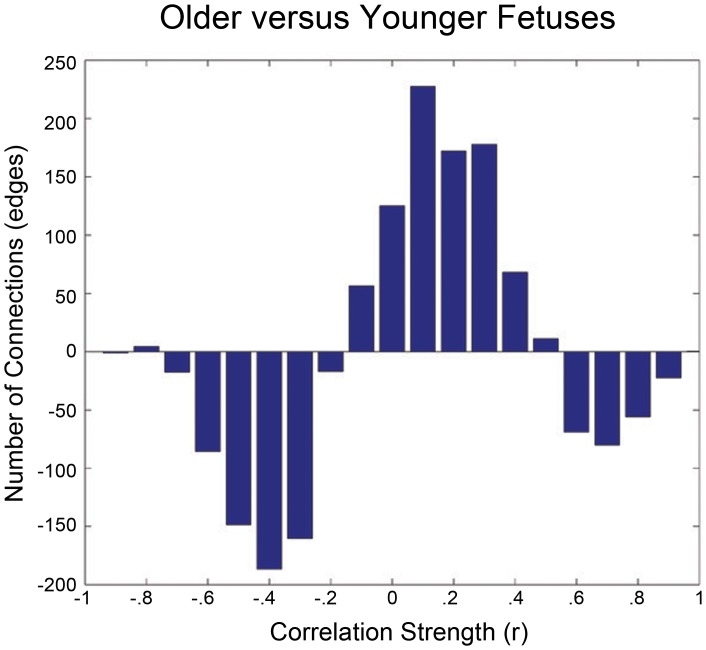
Range and frequency of functional correlations (r) in younger and older fetuses. A bar plot of younger fetuses’ correlation values subtracted from older fetuses’ correlation values is shown. The majority of correlation values measured in older fetuses occurred between -.1 and.5. In contrast, a wider range of correlation values were measured in younger fetuses. Differences in the full range of correlation values between young and old groups showed a trend, r = −0.32, p = 0.07.

The strength of functional connectivity was positively related to distance between long-range (top 5%) brain areas in older but not younger fetuses. In older fetuses, the longest of the long connections (Euclidean distance >73.5 mm) were most strongly correlated (p = 0.004; mean strength (r) of long connections = 0.14). This did not appear to be a motion related artifact [35], [36], because this comparison remained significant even when controlling for motion (r = 0.40, p = 0.02). No relationship was observed within the shortest connections (bottom 5%) for the older or younger fetal groups. Additionally, groups did not differ from one another in average strength of connectivity when assessing all possible connections. We did, however, observe a tighter range of pairwise correlation values with age, r = 0.35 p = 0.05, such that older fetuses showed correlation values (r) that were predominantly between −0.1 and 0.5 and younger fetuses showed a wider range of pairwise correlation values, see **Figure 4**.

### Negative Connectivity of the Posterior Cingulate Cortex (PCC)

**Figure 5:**
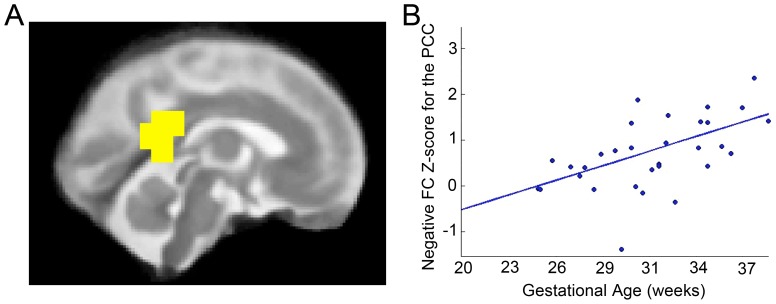
Negative connectivity in the posterior cingulate cortex (PCC). With advancing gestational age, intramodular anticorrelations of the PCC (in yellow, A) significantly increase (B), p<.05, FDR corrected. The PCC thus becomes more anticorrelated with other brain modules as fetuses mature.

Intermodular negative strength Z-score for the PCC was significantly positively related to age (robust regression t = 3.5 p<0.002). That is, the fetal PCC module demonstrated more negative connection to other brain modules with advancing GA, as depicted by the positive regression line in **Figure 5**. This was the only region with this significant effect. Based on the finding that more negative intermodular functional connectivity of the PCC occurs with age, we performed an exploratory search for functional connections from the PCC that became more negative with age. One region that was in a different module than the PCC consistently across participants, the right lateral posterior cerebellum, also demonstrated a significant negative correlation of age and functional connectivity strength with the PCC (r = −0.35, p = 0.04). Other significant age correlations with regional graph theory measures (robust regression, p<0.002) included negative strength (or the sum of all negative connections to a given node): left middle peri-rolandic parietal lobe (t = −3.42) and left dorsal posterior cerebellum (t = −4.81); negative participation coefficient: right inferior occipital lobe (t = 3.41); positive within-module strength z-score: left parietal lobe (t = 3.78); and positive between-module strength z-score: left lateral peri-rolandic parietal lobe (t = −3.64) and right inferior occipital lobe (t = 3.66).

## Discussion

This study demonstrates the utility of fetal fMRI for discovering principles of neural system organization at the beginning of human life. Our results indicate the fetal brain is organized with modular structure, wherein connections are much stronger within than between modules. This is in agreement with observations in adults [37], and suggests modularity is an early emergent characteristic of the developing brain. We also found that with advancing gestational age intermodule connection strength and negative connectivity between the PCC and other brain regions increase. This study applied graph theoretical techniques, which are favorable for evaluation of fetal brain functional organization where *a priori* knowledge is limited.

Reduced intermodular connection strength and high modularity in younger fetuses suggest that in early fetal life functional systems are independent, and only with time do these begin to collaborate more fully as members of a whole brain system. Prior observations in late childhood, adolescence, and adulthood have provided mixed evidence about age-related independence of brain modules. Early research demonstrated that brain modules become increasingly independent and separable with advancing age [21], [38]. However, recent fcMRI research in large (N>100), motion-corrected samples describe linear decreases in modularity with age in participants ranging from ages 7 to 85 years [39]. The apparent contradiction in these results may relate to experimental approach differences. In particular, the latter performed analyses across a wide age range, which may favor identification of life-span related patterns of change. Evaluation of brain modules in tighter age ranges may be more sensitive to developmental transitions, and these may show transitions from childhood to young adulthood are characterized by increased brain modularity. Our research extends the age range covered in these prior works by many years to provide the first evidence that modularity decreases in the prenatal brain. It is possible that development of functional circuitry in the antenatal brain proceeds in an inverted-U [40]. During pre- and post-natal life functional modules may develop, become integrated (possibly over integrated) then with development these may be pruned, or trimmed back, and functional substructures may become, again, more separable. The major difference apparent on either side of this proposed U is that the modules observed here in early fetal life are spatially restricted (i.e., dense local connectivity), whereas the modules observed in the shift to young adulthood span long distances in the brain, facilitating communication between widely dispersed regions [15], [21], [41].

Here, we observe that modules obtained from graph analysis of the human fetal brain comprise areas that will later support vision, movement, language, and data integration (e.g., multimodal, association cortices). For example, we identified a module that spans somatosensory and motor cortices, resembling a network obtained by clustering analysis in a large (n = 1000) adult functional connectivity dataset [42]. It appears that these systems become integrated during fetal life, and remain collaborative throughout the life span. Further, we noted a module that included regions of the default mode network (DMN) as defined in the extant neuroimaging literature [43]. Discovering coordinated DMN function *in utero* is consistent with studies in term- and preterm-born infants indicating that a ‘proto-default-mode network’ is established in early life [44], [45]. We suggest that modules observed in fetuses may constitute developing functional systems, and acknowledge further work is needed to track the emergence of the default mode and other functional networks.

While similar brain networks (i.e., modules) were identified in older and younger fetuses, their spatial distributions differed. For instance, a module that largely corresponded to left lateralized language regions appeared to encompass Broca’s and Wernicke’s Areas in older but not younger fetuses. In addition, the cerebellum was included in a module that contained sensorimotor regions in older but not younger fetuses. This is consistent with fcMRI studies of adults that demonstrate intrinsic functional connections between motor and cerebellar regions [46], reflecting underlying anatomical connectivity of these regions via thalamic pathways. The present work extends these observations by providing provisional maps of evolving neural connectivity systems present in fetal life.

Two observations in these data converge in support of a central role for the PCC in the development of functional brain networks in fetal life. First, we observed increased negative FC between the PCC and other brain regions with advancing gestational age (**Figure 5** **)**. Second, we found that PCC/superior parietal regions (**Figure 3**, green) were functionally connected to regions of the prefrontal cortex in older fetuses only. Previous studies of both functional and structural networks have demonstrated the PCC is a major cortical connectivity hub [7]–[9], even in infancy [18]. The PCC is also a primary hub of the DMN [47]. This network, comprised of PCC, medial prefrontal, and lateral parietal regions, has an anticorrelated relationship with the dorsal attention network [48]–[50], and this dynamic relationship is compromised in disease [51], and mediates adaptive cognitive function [52], [53]. We present evidence that the negative opposition of the PCC to other brain networks begins to be established in human fetal life. Early and emergent anti-correlated function between the PCC and other brain regions suggests the PCC may serve a foundational role in the establishment of functional neural networks in the human fetal brain.

Methodological considerations of the current study warrant discussion. A chief consideration is that neurovascular coupling and cerebrovascular reactivity are not well understood in the human fetal brain. The BOLD fMRI connectivity approach is reliant upon reverse inference about the development of neural networks from patterns depicted by blood oxygen signals. Uncertainty about neural mechanisms that give rise to fMRI correlations is not unique to fetal imaging (see review by [54]), but immaturity of the fetal brain presents an added complexity. Available evidence from the adult brain indicates that functional connectivity is most highly correlated with neural local field potentials and gamma range activity [54], [55]. Considerations in the current study are that physiological properties underlying BOLD hemodynamic signals in our early and late fetuses may differ, and that we lack knowledge about how fetal BOLD hemodynamics *in utero* compare to postnatal hemodynamics. Despite evidence that BOLD fMRI hemodynamic responses are reliably identified in infants born prematurely [56], rodent studies show positive BOLD response is not detected in the rat brain until postnatal day eleven [57], which equates to approximately 28 weeks in human gestation. However, observations about the directionality of BOLD activity do not have direct bearing on conclusions drawn from the current data, as BOLD *activity* must not be confused with BOLD *connectivity*. It is possible that BOLD connectivity analysis of the present study is more robust to developmental changes in hemodynamic response properties than would a task based fMRI activation map, by comparison. We observed a trend for functional correlations to become more positive (shifting from more negative) and to have a more narrow range in distribution with increasing fetal age (**Figure 4**). It is possible that these features depict important properties within development of neurovascular reactivity in human fetuses that are presently not known. With increased use of this methodology in human fetuses there will certainly be a need for increased study of the neurovascular elements of the obtained measures.

Another methodological consideration for the current work is the significant problem of fetal movement. Conventional approaches for motion correction cannot adequately address the magnitude and conditions of movement encountered in fetal fMRI. For example, because movements of the fetus are independent of the mother, but both are included in the acquired volume, standard tools for plotting, thresholding, or correcting movement are rendered ineffective. We have taken an approach in this and our prior work [2] that requires manual extraction of the fetal brain and realignment of volumes to the first volume of each protracted period of minimal movement, for which standard motion correction procedures are adequate. However, because younger fetuses move more than older fetuses, this approach resulted in significantly more analyzed frames in older fetuses. It may be useful for future studies to exclude even greater quantities of data in older fetuses so to constrain groups to having no difference in total number of volumes analyzed. We report loss of 39% of fMRI volumes to movement in this fetal sample. While this is extreme, (1) longer scans were collected in anticipation of this effect, leaving us with sufficient data of fair quality, and (2) this degree of data loss is similar to studies of adults that used stringent movement thresholds (35 to 39% [35]). As fetal fMRI is in nascent stages, considerable research remains to develop best practices for addressing the magnitude of movement and unique challenges inherent in fetal fcMRI methodology. While these methodological caveats make fetal FC more challenging to interpret, fcMRI remains a powerful new technique for non-invasively detecting neural connections *in utero*.

This study summarizes observations about development of brain networks prior to birth in healthy human fetuses. We found that as human fetuses advance in gestational age, intermodule connection strength increases, modularity decreases, modules begin to overlap with known functional systems, and function in the PCC becomes more negatively correlated with other brain areas. Understanding brain development in fetal life has relevance for both human neuroscience and for understanding the brain basis of common neurological problems that may not be observable using other imaging approaches. Neuroimaging research shows, for example, that brain maturation differs between the sexes [58], and that infants born prematurely have altered neuroconnectivity [59], but we have no knowledge about whether these differences emerge prenatally. There is a need to further develop fetal connectivity fMRI methodology and to apply this technique to the study of human fetal health and disease. Establishment of a new field of fetal fcMRI will provide an unprecedented means for evaluating brain development at the beginning of life, and will enable a platform for discovering the fetal origins or antecedents of neural injury or disease.
